# Supplementary figures and images for: Prediction of sporulating Firmicutes from uncultured gut microbiota using SpoMAG, an ensemble learning tool
Source: PeerJ. 2025 Oct 17;13:e20232. doi: 10.7717/peerj.20232 (PMC12536801; doi:10.7717/peerj.20232)

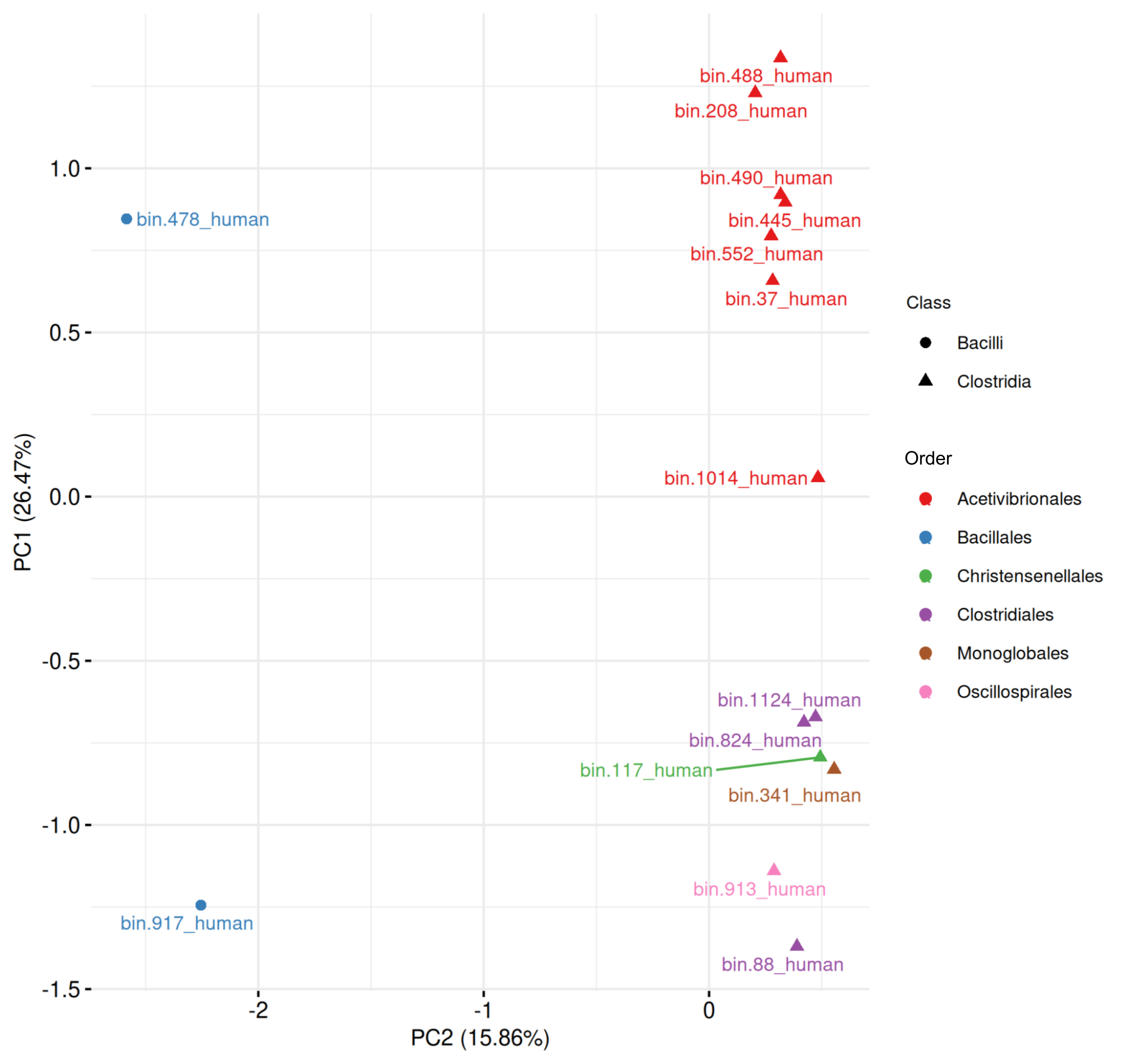

Supplement: Supplemental Information 1 [file peerj-13-20232-s001.png]

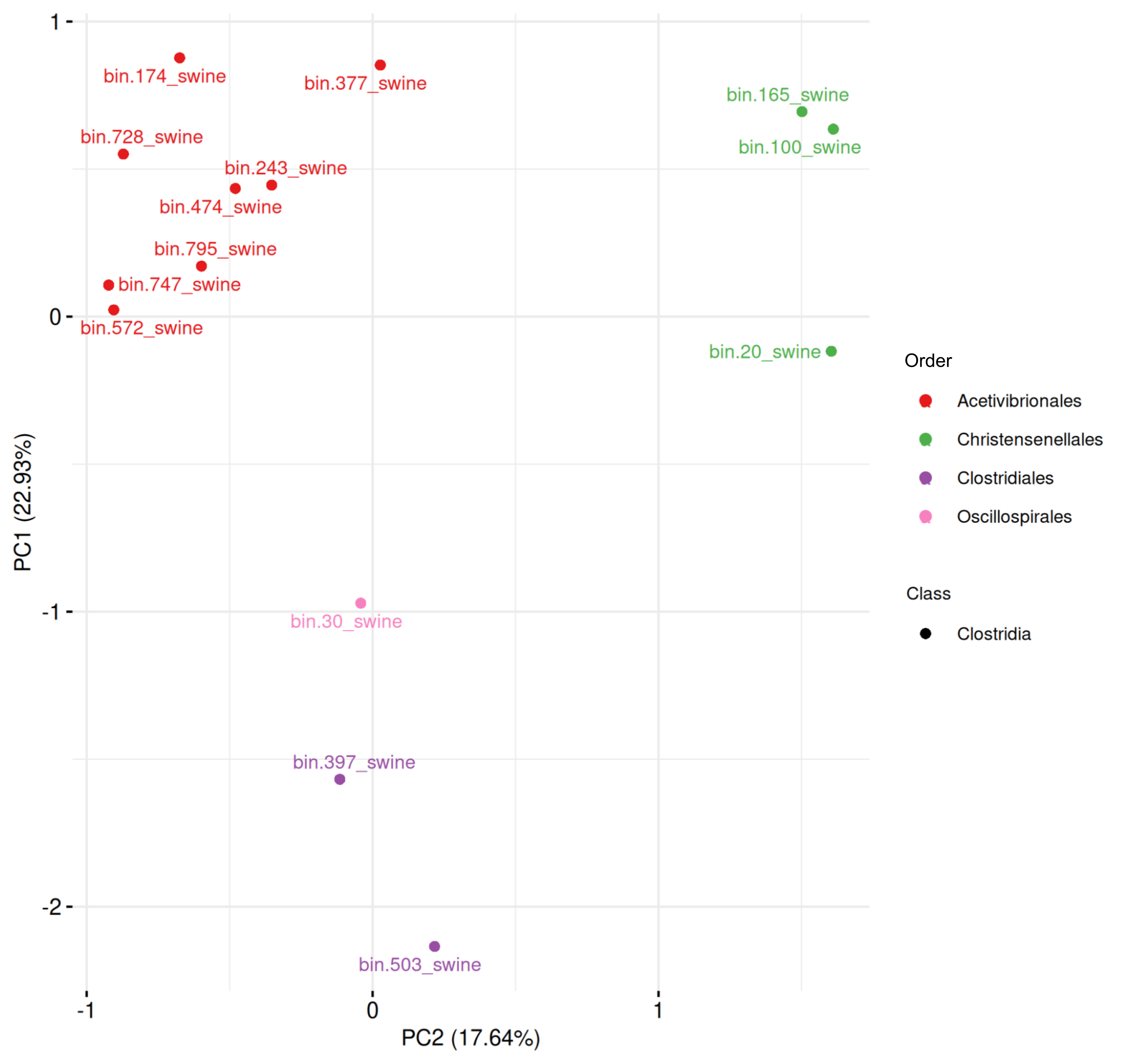

Supplement: Supplemental Information 2 [file peerj-13-20232-s002.png]

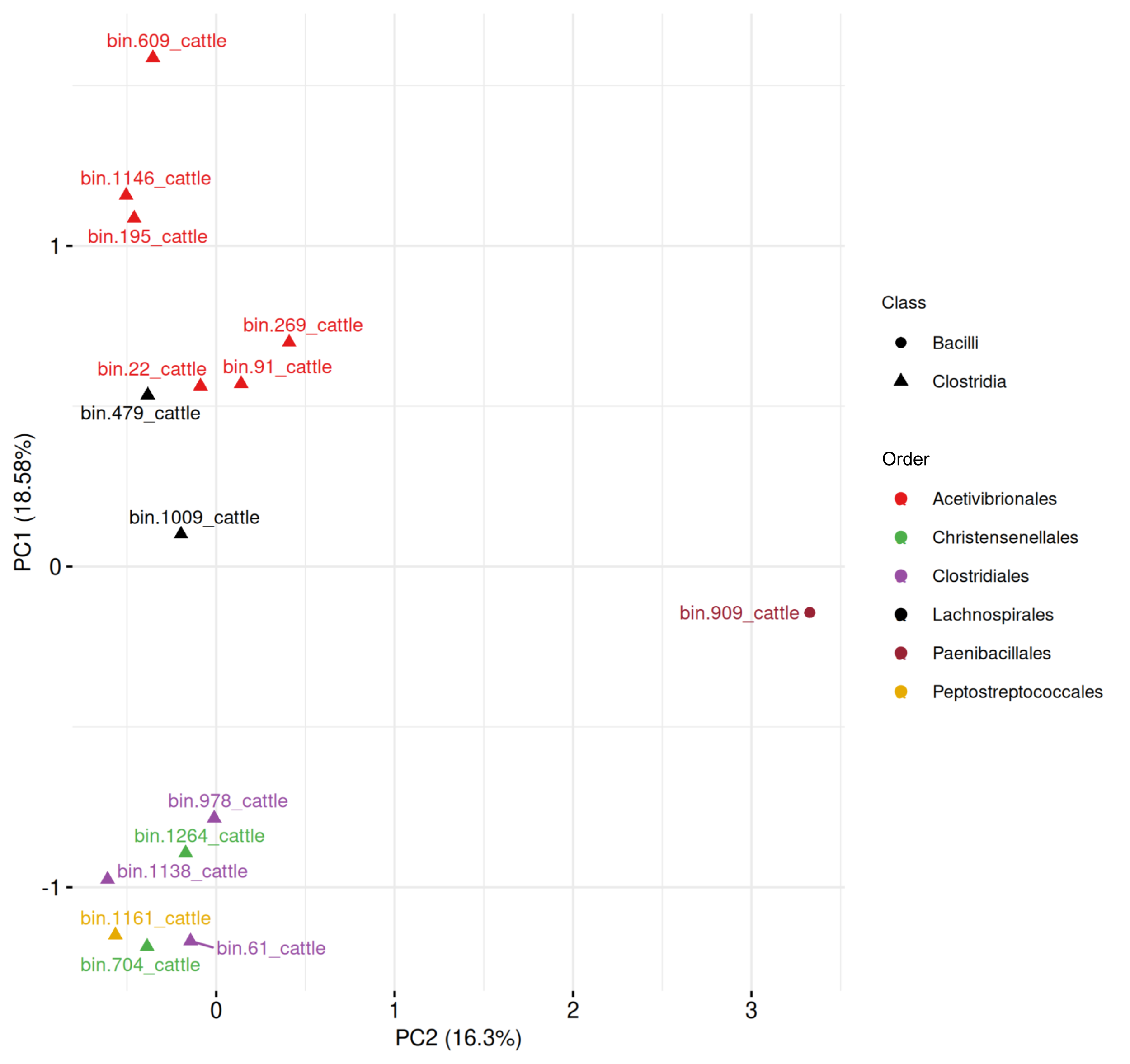

Supplement: Supplemental Information 3 [file peerj-13-20232-s003.png]

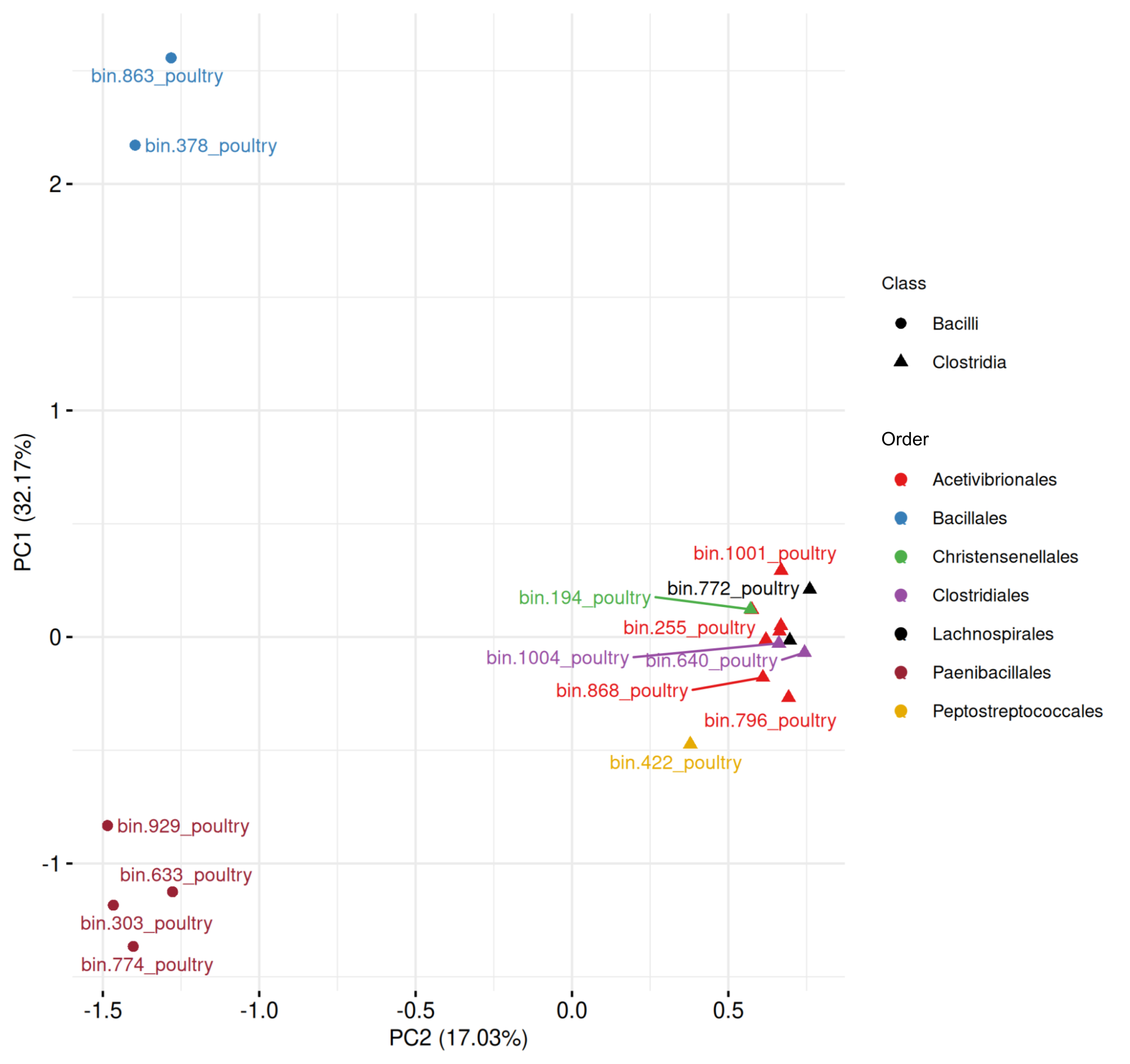

Supplement: Supplemental Information 4 [file peerj-13-20232-s004.png]
